# Dellaglioa Algida Cell-Free Supernatant Inhibits Pseudomonas Fluorescence and Pseudomonas Fragi by Destroying Cell Membranes

**DOI:** 10.3390/foods13182986

**Published:** 2024-09-20

**Authors:** Yao Sun, Tianhui Wei, Tongqing Ma, Zhiying Fan, Jinzhu Song

**Affiliations:** School of Life Science and Technology, Harbin Institute of Technology, Harbin 150001, China; sunyao@stu.hit.edu.cn (Y.S.); weitianhui@stu.hit.edu.cn (T.W.); mtq912@stu.hit.edu.cn (T.M.); 23s028029@stu.hit.edu.cn (Z.F.)

**Keywords:** *Lactobacillus*, cell-free supernatant, inhibition, *Pseudomonas*

## Abstract

The aim of this study was to examine the components of the cell-free supernatant (CFS) derived from a novel strain of psychrophilic *Lactobacillus*, *Dellaglioa algida*, and to further elucidate the impact of this CFS on various cellular processes. Specifically, we sought to understand its effects on the cell membrane, protein and DNA release, protease activity, and metabolites of *Pseudomonas fluorescens* and *Pseudomonas fragi*, thereby clarifying the antibacterial mechanism involved. The CFS components were analyzed using Gas Chromatography–Mass Spectrometry (GC-MS), the Coomassie Brilliant Blue method, and the phenol–sulfuric acid method. The inhibitory effect of the CFS on *Pseudomonas fluorescens* and *Pseudomonas fragi* was assessed using the ethidium bromide (EB) assay, Oxford cup assay, and ultramicroassay. Additionally, we analyzed the metabolites produced by *Pseudomonas fluorescens* and *Pseudomonas fragi* when treated with the CFS. The findings reveal that the CFS of *Dellaglioa algida* contains 94 volatile components, with protein and sugar concentrations of 32.857 ± 0.9705 mg/mL and 98.250 ± 4.210 mg/L, respectively. The CFS induces varying degrees of damage to the cell membranes of both *Pseudomonas fluorescens* and *Pseudomonas fragi*, leading to the release of intracellular proteins and DNA. Furthermore, the CFS reduced the protease activity and metabolic capacity of *Pseudomonas fluorescens* and *Pseudomonas fragi*. These results enhance our understanding of the mechanism by which psychrophilic *Dellaglioa algida* inhibits *Pseudomonas fluorescens* and *Pseudomonas fragi*, confirming that its inhibitory effect predominantly occurs through damage to the biological cell membranes of *Pseudomonas*. *Dellaglioa algida* is a newly identified cold-adapted inhibitor of *Pseudomonas*, indicating that its CFS is an effective microbial inhibitor in cold environments. This discovery suggests potential applications in inhibiting the growth and reproduction of *Pseudomonas fluorescens* and *Pseudomonas fragi* in food, pharmaceuticals, perfumes, and other chemicals, providing a valuable new reference for industrial preservation.

## 1. Introduction

Biological inhibitors have been receiving increasing attention, as traditional chemical inhibitors frequently alter the properties of food and drugs. The cell-free supernatants (CFSs) of *Lactobacillus*, which is a genus with widespread probiotic species, have garnered significant attention by virtue of their unique sensory attributes and superior physiological activity. *Lactobacillus* species produce a diverse range of metabolites, including organic acids, proteins, sugars, phenolic compounds, aldehydes, and alkanes [1]. The varying properties of these substances yield CFSs with different physiological activities, such as antibacterial [2], antioxidant [3], anti-inflammatory, and anti-infective effects [4]. Thus, the investigation of the metabolites of *Lactobacillus* and the corresponding mechanisms is likely to contribute to the development of *Lactobacillus*-derived food additives, drugs, perfumes, or other chemistry-related applications.

Numerous studies have demonstrated the potent antibacterial properties of CFSs derived from *Lactobacillus*, which exhibit inhibitory effects on both fungi and bacteria. For instance, probiotic CFSs obtained from *Lactobacillus crispatus*, *Lactobacillus gasseri*, and *Lactobacillus vaginalis* can inhibit budding, hyphae, and biofilm formation by *Candida albicans* [5]. The CFS of *Lactobacillus gasseri* 63 AM has been found to impede the growth of the *Pseudomonas aeruginosa* PAO1 strain, thwart its formation of biofilms, and partially eradicate pre-existing ones [6]. *Lactobacillus crustorum* ZHG-1-1, isolated from traditionally pickled cucumbers, has been identified as having the potential to inhibit virulence factors regulated by Quorum Sensing (QS) and biofilm formation by the foodborne pathogen *Pseudomonas aeruginosa* [7]. Hence, the inhibitory effect of *Lactobacillus* on bacteria or fungi may be related to disturbance or damage to the formation of the cell membrane, and the detailed mechanisms of different *Lactobacillus* in their working environments are worthy of further investigation for their potential in the food, drug, and chemical industries.

The components in the CFSs of *Lactobacillus* have been examined by numerous researchers; however, instead of investigating further relations between CFS components and cell membrane damage, they focused on analyzing the inhibitory effects of *Lactobacillus* CFSs on pathogens. Specifically, the CFS of *Lactobacillus plantarum* has been found to reduce the pathogenic bio-burden, necrotic tissue quantity, and wound area while promoting wound cleaning, granulation tissue, and wound healing, where the primary active ingredients include the lactone of hydroxyvaleric acid, 5-methyl-hydantoin, and benzoic acid [8]. *Lactobacillus reuteri* demonstrates antibacterial activity against *Pseudomonas fluorescens* and *Pseudomonas putida*, both of which are isolated from milk microbiota in dairy processing facilities [9]. One can conclude that the CFSs of *Lactobacillus* are emerging safe and effective microbial preparations that hold promise for controlling the growth and reproduction of pathogenic and spoilage bacteria during food and drug processing and transportation. However, the possible relationship between damage to the cell membrane and the competence of the CFS has not been investigated yet.

Moreover, almost all of the abovementioned studies on antibacterial *Lactobacillus* CFSs were conducted at room temperature—that is, they showed antibacterial activity against pathogens at room temperature. However, *Pseudomonas fluorescens* [10,11,12,13] and *Pseudomonas fragi* [13,14]—both conditionally pathogenic bacteria that can pose risks to food and drugs—demonstrate strong adaptability to cold environments. Common *Lactobacillus* CFSs that work at room temperature have little antibacterial effect on pathogenic bacteria that are active in low-temperature environments, such as those in transportation and storage, mainly because *Lactobacillus* species that survive at low temperatures are relatively rare and their physiological activity is very different from that of common *Lactobacillus*.

The bacterium *Lactobacillus algidus*, originally isolated from vacuum-packed frozen beef by Y. Kato in 2000 [15], was reclassified as *Dellaglioa algida* in 2020 [16,17,18]. This facultative anaerobic, psychrophilic, non-motile, homofermentative, rod-shaped bacterium [19,20] is a highly rare variant of the psychrophilic *Lactobacillus* species. As a novel strain of *Lactobacillus*, its fermentation products likely possess unique components and effects that enable it to adapt more effectively to cold environments. Concurrently, *Pseudomonas fluorescens* [10,11,12,13] and *Pseudomonas fragi* [13,14]—both conditionally pathogenic bacteria that can pose risks to food and drugs—also demonstrate strong adaptability to cold environments. Therefore, exploring the antibacterial effect of *Dellaglioa algida*, which has psychrophilic properties, in the abovementioned cold environments will provide a new solution for inhibiting pathogenic bacteria in cold environments, with potential application prospects in the fields of cold-chain transportation of food and medicine.

Motivated by the above observations, the objectives of this study were to investigate the components of the *Dellaglioa algida* CFS and its inhibition mechanism with regard to *Pseudomonas fluorescence* and *Pseudomonas fragi* cell membrane destruction. We examined the volatile compounds, proteins, and sugar content in the CFS of *Dellaglioa algida* by utilizing techniques such as Gas Chromatography–Mass Spectrometry (GC-MS). Then, we scrutinized the impact of the CFS on cell membrane damage, protease activity, protein and DNA release, and variations in the metabolites of *Pseudomonas fluorescens* and *Pseudomonas fragi* by employing methods including EB staining, the milk plate method, ultra-trace substance analysis, and GC-MS. The results in this paper will provide new references for the development of probiotic-derived preparations that inhibit cold-resistant pathogenic bacteria and can be utilized in the food, drug, and chemistry industries.

## 2. Materials and Methods

### 2.1. Bacterial Strains, Media, and Culture Conditions

*Dellaglioa algida* was cultured in de Mann–Rogosa–Sharpe (MRS) broth purchased from Biosharp^®^ at 20 °C for 24 h without agitation to generate the seed liquid of *Dellaglioa algida*, following methods primarily cited by Elina et al. [20,21]. The activated seed liquid of the *Dellaglioa algida* strain was then inoculated into the liquid MRS medium with a bacterial load of 1% (*v*/*v*) and subsequently activated twice at 20 °C for 24 h in each instance. Following activation, the bacterial suspension underwent centrifugation (8000× *g*, 5 min, 4 °C) and sterile filtration using low-protein-binding cellulose acetate membrane filters with a pore size of 0.22 μm to yield the CFS. After freeze-drying, the CFS was preserved at −80 °C for future use.

The bacteria *Pseudomonas fluorescens* (BNCC^®^ 335852^TM^) and *Pseudomonas fragi* (BNCC^®^ 134017^TM^), purchased from BeNa Culture Collection (BNCC), China, were cultivated in Tryptone Soy Broth (TSB) medium purchased from Biosharp^®^ at a temperature of 28 °C with a shaking speed of 180 rpm/min for a duration of 24 h to produce the seed liquid. This activated seed liquid was subsequently inoculated into TSB medium, maintaining a 1% (*v*/*v*) inoculum size, and allowed to activate for an additional 18 h at the same temperature before being used in subsequent experiments.

### 2.2. Gas Chromatography–Mass Spectrometry Analysis of Total Composition of CFS

The extraction method involved subjecting the 10 mL CFS sample to two extractions with an equal volume of ethyl acetate, which contained 0.1% acetic acid, followed by a one-hour settling period. Subsequently, the ethyl acetate extract was evaporated through rotary evaporation for 40 min at a temperature of 35 °C to eliminate the solvent. The resulting residue was dissolved in 1 mL of methanol and filtered through a 0.22 μm membrane filter. This solution was then stored at −80 °C for future use.

The chromatographic analysis was performed using an Agilent HP-5MS column (Agilent 19091S-133, Beijing, China, 30 m × 250 μm × 0.5 μm). The carrier gas utilized was helium with a flow rate of 1 mL/min. The injection port temperature of the GC-MS instrument was set to 200 °C. The temperature program commenced at an initial temperature of 50 °C, escalating at a rate of 40 °C/min to reach 50 °C. This was followed by a further increase at a rate of 50 °C/min up to 250 °C and, finally, a slow increase at a rate of 0.5 °C/min until reaching 300 °C. The transfer line temperature was maintained at 250 °C. The sample injection volume was precisely controlled at 1 μL with a split ratio of 50:1. The solvent delay and total run time were set at 3 min and 18 min, respectively. The mass spectrometry detection conditions included an electron energy of 70 eV, an ion source temperature of 230 °C, and a quadrupole temperature of 150 °C. The total substance analysis of the CFS was carried out in full-scan mode, ranging from *m*/*z* 15 to 800.

### 2.3. Protein Content Test of CFS

Coomassie Brilliant Blue, a dye that appears red in its free state and blue when bound to proteins, has a maximum absorption peak at 595 nm. Consequently, a colorimetric method was utilized to quantitatively ascertain the protein content in the CFS [22]. Bovine serum albumin (BSA) served as the standard. Solutions of the CFS at a concentration of 5 mg/mL and BSA solutions at concentrations ranging from 20 to 200 μg/mL were prepared. Each solution was added in triplicate to a 96-well plate, with 100 μL of each solution being used. Subsequently, 100 μL of Coomassie Brilliant Blue reagent was added to each well followed by incubation at room temperature for 5 min. The absorbance at 595 nm was measured using a microplate reader. A standard curve was constructed with absorbance on the vertical axis and BSA concentration on the horizontal axis. The protein content in the samples was then calculated based on the linear relationship between the absorbance and BSA concentration.

### 2.4. Test of Content of Total Sugars in CFS

The content of total sugars in the CFS was determined using the phenol–sulfuric acid method [23]. A precisely weighed 0.05 g sample of glucose, dried to a constant weight, was placed in a small beaker and dissolved in deionized water. This solution was then diluted to 100 mL in a volumetric flask to prepare a 0.5 mg/mL glucose standard solution. Then, 6 g of phenol was accurately weighed, dissolved in distilled water, and diluted to 100 mL in a volumetric flask. The mixture was thoroughly mixed and set aside. Following a gradient of 0–0.25 mg/mL, the glucose standard solution and distilled water were added to 1 mL of the phenol solution. After thorough mixing, concentrated sulfuric acid was rapidly added. The mixture was shaken and incubated in a 100 °C water bath for 60 min. Upon removal, it was quickly cooled to room temperature, and the absorbance was measured at 490 nm. Next, 100 mg of CFS powder was accurately weighed and dissolved in distilled water to make up 20 mL. The absorbance value was then measured. By substituting these values into the equation, the content of total sugars was obtained.

### 2.5. Effect of CFS on Viability of Pseudomonas fluorescens and Pseudomonas fragi

Overnight cultures of *Pseudomonas fluorescens* and *Pseudomonas fragi* were activated in a shaker at 28 °C. The cultures were then inoculated (1%) into test tubes containing 5 mL of medium, followed by incubation for 6 h to reach the logarithmic growth phase. CFS powder was added to achieve final concentrations of 0, 0.5, 1.0, and 2.0 mg/mL, and the mixture was incubated overnight at 28 °C. EB powder was dissolved in deionized water (or ultrapure water) to prepare a 10 mg/mL solution. After it was fully dissolved, it was stored at room temperature or 2–8 °C away from light. The ethidium bromide (EB) staining solution was diluted 50–100 times with PBS according to the number of samples to prepare the EB staining working solution. A total of 50 μL of the bacterial suspension was combined with 2 μL of the EB working solution and incubated at room temperature for 5–15 min. Clean microscope slides were prepared, onto which 5–10 μL of the bacterial suspension was dropped, covered gently, and observed under a fluorescence microscope with an excitation wavelength of 485 nm, observation and photography at 630 nm, and a magnification of 400×. Then, Image J was utilized to analyze the damage extent of the cell membranes of *Pseudomonas fluorescens* and *Pseudomonas fragi*, which was quantified by the content of DNA released and protein leakage.

### 2.6. Effect of CFS on Protease Activity of Pseudomonas fluorescens and Pseudomonas fragi

Sterile skim milk (10 mL) was added to 90 mL of LB medium, which had been cooled to approximately 50 °C, and rapidly mixed before being poured into Petri dishes. These were then left to solidify, forming skim milk plates with Oxford cups punctured in them. Once the medium had solidified, 100 μL of the supernatant from *Pseudomonas* treated with varying concentrations (0.5, 1.0, 2.0 mg/mL) of the CFS was introduced. Untreated *Pseudomonas fluorescens* and *Pseudomonas fragi* supernatants served as controls, while 4.0 mg/mL papain was used as a positive control. Following the incubation of the plates in a sterile chamber for 24 h, the sizes of the transparent zones were measured, with at least three replicates conducted for each group. Then, the relative protease activity was calculated as follows: relative protease activity = control group − treatment group/control group × 100%.

### 2.7. Effect of CFS on Amount of Protein and DNA Released from Pseudomonas fluorescens and Pseudomonas fragi

*Pseudomonas fluorescens* and *Pseudomonas fragi* were activated overnight in a shaker at 28 °C, inoculated at 1% into test tubes containing 5 mL of medium, and then cultured for 6 h until the logarithmic growth phase. Subsequently, CFS powder was added to achieve final concentrations of 0, 0.5, 1.0, and 2.0 mg/mL, followed by overnight incubation at 28 °C. The bacterial suspension was centrifuged (8000× *g*, 10 min, 4 °C), and the supernatant was collected after filtration through a 0.45 μm membrane filter. The protein and DNA released in the supernatants of *Pseudomonas fluorescens* and *Pseudomonas fragi* were measured using a microplate spectrophotometer, with 6 replicates for each group.

### 2.8. Effect of CFS on Composition of Pseudomonas fluorescens and Pseudomonas fragi Metabolites

The extraction procedure was identical to that described in Section 2.2. An Agilent HP-5MS chromatographic column (Agilent 19091S-133, 30 m × 250 μm × 0.25 μm) was employed with helium as the carrier gas at a flow rate of 1 mL/min. The injection port temperature of the GC-MS instrument was set to 200 °C and then increased to 220 °C at a rate of 10 °C/min, followed by an increase to 250 °C at a rate of 5 °C/min and finally to 252.5 °C at a rate of 0.5 °C/min. The transfer line temperature was held constant at 280 °C. A sample volume of 1 μL was injected using split injection with a split ratio of 50:1. The solvent delay and total run time were set to 3 min and 18 min, respectively. The mass spectrometry detection conditions were as follows: electron energy was set to 70 eV, and the temperatures of the ion source and quadrupole were maintained at 230 °C and 150 °C, respectively. The ion monitoring mode (*m*/*z* 143) and full-scan mode (*m*/*z* 15,800) were utilized for the analysis and detection of the total substances in the supernatants of *Pseudomonas fluorescens* and *Pseudomonas fragi*.

### 2.9. Data Analysis

The data collected from at least three independent trials are represented as mean values with standard deviations. Statistical significance, denoted by * *p* < 0.05, ** *p* < 0.01, and *** *p* < 0.001, was analyzed via one-way ANOVA and a T-test using GraphPad Prism 7 and Origin 2018 software. Additionally, the GC-MS data were cross-referenced with the NIST 7 database.

## 3. Results

### 3.1. Composition of CFS

**Figure 1:**
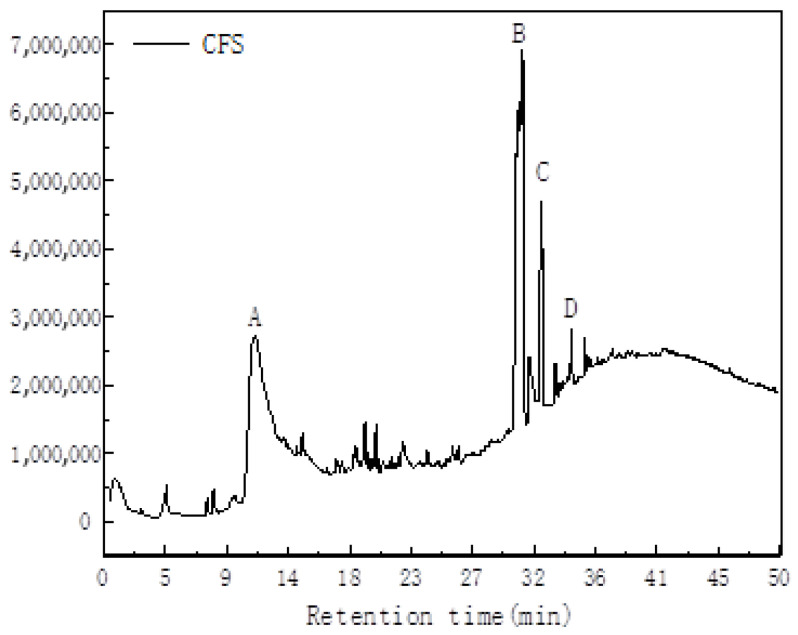
Analysis of total composition of CFS using GC-MS.

Upon comparison with the NIST 7 database, a total of 94 substances were identified in the CFS. These included butyric acid, valeric acid, benzoic acid, and phthalic acid, which constituted a significant proportion of the acids detected. Additionally, the presence of aldehydes, phenols, and other organic compounds was noted, along with smaller molecules such as urea and cyclopentane. As illustrated in Figure 1, Peak A was determined to be lactone 4-Heptanone, exhibiting a peak area of 2,937,043, an A/H ratio of 11.59, and a content ratio of approximately 31.56%, thereby establishing itself as the ketone with the highest proportion. Peak B was identified as Cycloheptasiloxane, characterized by a peak area of 271,905, an A/H ratio of 1.55, and a content ratio of approximately 2.92%. Peak C was identified as valeric acid, demonstrating a peak area of 4,748,302, an A/H ratio of 2.63, and a content ratio of approximately 51.03%, thus emerging as the compound with the highest content ratio in the fermentation broth. Finally, Peak D was identified as 2,5-Dihydroxybenzoic acid, a 3TMS derivative, with a peak area of 199,037, an A/H ratio of 1.04, and a content ratio of approximately 2.14%.

**Figure 2:**
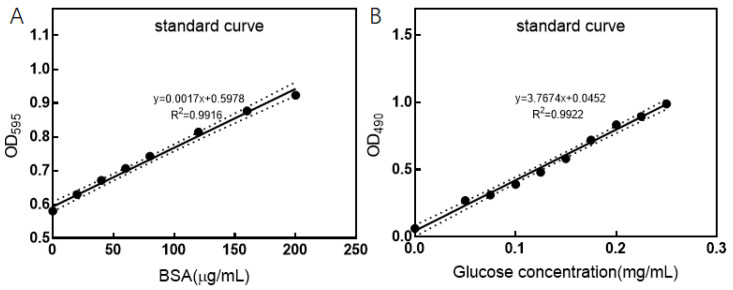
Standard curve of BSA and glucose concentration (**A**): standard curve of BSA; (**B**): standard curve of glucose concentration).

Figure 2A depicts a linear relationship between the concentration of bovine serum albumin (BSA), as determined by the Coomassie Brilliant Blue method, and the absorbance at ${\mathrm{OD}}_{595}$, represented by the equation y = 0.0017x + 0.5978, with an $R^{2}$ value of 0.9916. The x-axis contains the concentration of BSA, while the y-axis represents absorbance. Utilizing this linear relationship, the protein content of the CFS is calculated to be 32.857 ± 0.9705 mg/mL. Figure 2B presents a linear relationship between the concentration of glucose, as measured by the phenol–sulfuric acid method, and the absorbance at ${\mathrm{OD}}_{490}$, expressed by the equation y = 3.7674x + 0.0452, with an $R^{2}$ value of 0.9922. The x-axis signifies the concentration of glucose in mg/mL, and the y-axis represents absorbance. Based on this linear relationship, the content of total sugars in the CFS is calculated to be 98.250 ± 4.210 mg/L.

### 3.2. Influence of CFS on Viability of Pseudomonas fluorescens and Pseudomonas fragi

**Figure 3:**
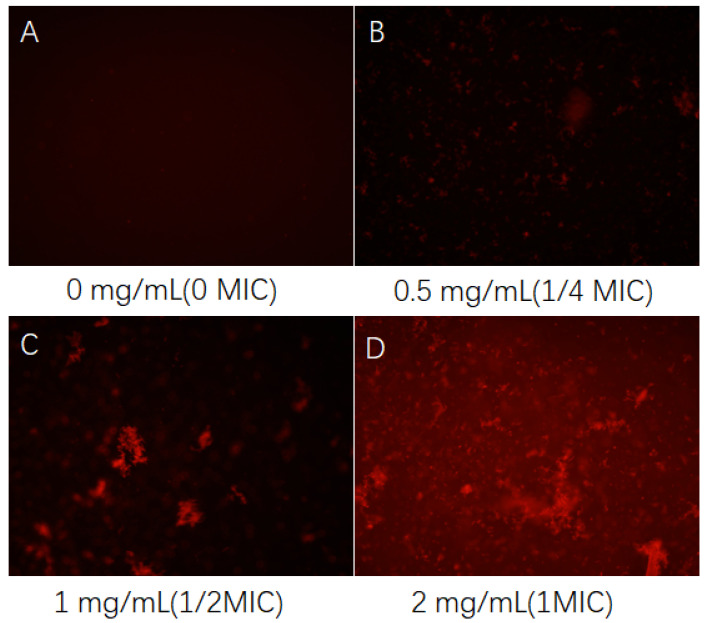
Effect of CFS on cell membrane damage in *Pseudomonas fluorescens*, where subfigures (**A**–**D**) stand for the cases of various concentrations.

As depicted in Figure 3, the cell membrane of normally growing *Pseudomonas fluorescens* remains intact, as evidenced by the failure of EB to penetrate it and emit fluorescence. However, upon the addition of 0.5 mg/mL CFS, red fluorescence emerges in the field of view, signifying bacterial death induced by the CFS. Moreover, when the concentration of the CFS increases to 1 mg/mL, a pronounced clustering of dead bacteria is observed. The electron microscopy results can be found in our previous study [21], which established that the CFS does not impact the biofilm of *Pseudomonas fluorescens*. This finding reaffirms that the inhibition of *Pseudomonas fluorescens* by the CFS is not attributed to the disintegration of the biofilm but rather to the formation of pores in the cell membrane, which results in bacterial death. When the fermentation broth concentration reaches 2 mg/mL, all bacteria are deceased, and the clustering of dead bacteria persists.

**Figure 4:**
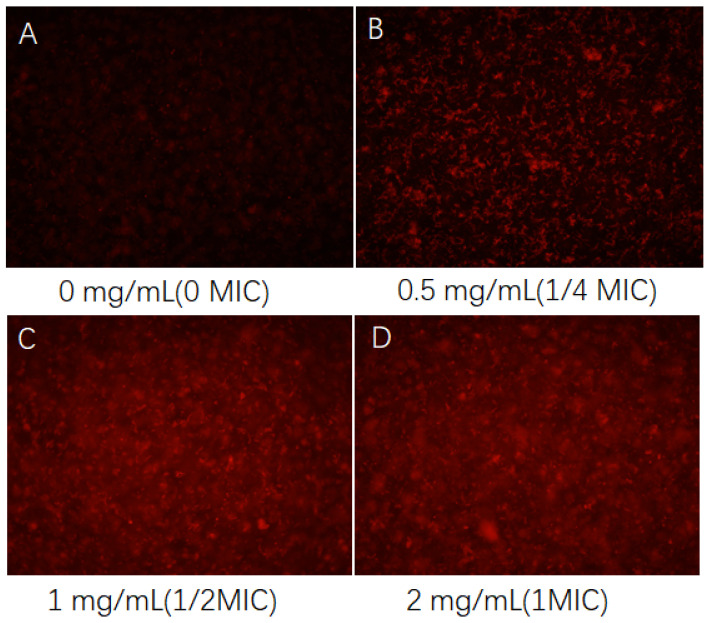
Effect of CFS on cell membrane damage in *Pseudomonas fragi*, where subfigures (**A**–**D**) stand for the cases of various concentrations.

As depicted in Figure 4, the cell membrane of normally growing *Pseudomonas fragi* remains intact, as evidenced by the failure of EB to penetrate it and emit fluorescence. However, upon the addition of 0.5 mg/mL CFS, red fluorescence emerges in the field of view, signifying bacterial death induced by the CFS. Moreover, when the concentration of the CFS increases to 1 mg/mL, a substantial amount of bacterial death is observed without any clustering. Previous research has suggested that the fermentation broth impedes the growth of *Pseudomonas fragi* biofilm [21]. This finding further implies that the inhibitory mechanism of the CFS on *Pseudomonas fragi* may encompass damage to both its biofilm and cell membrane, resulting in bacterial death. When the concentration of the CFS reaches 2 mg/mL, all bacteria are deceased, with no clustering of dead bacteria observed.

### 3.3. Effect of CFS on Extracellular Protease Activity of Pseudomonas fluorescens and Pseudomonas fragi

**Figure 5:**
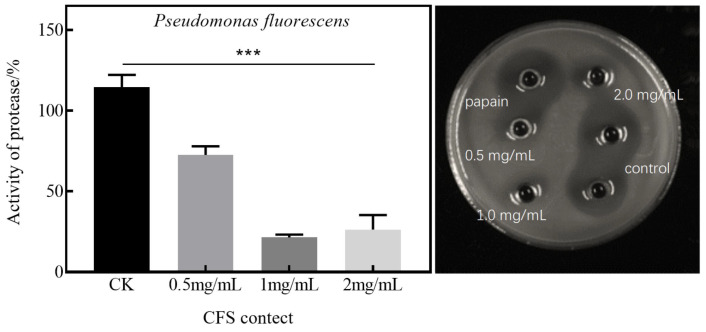
The effect of the CFS on the extracellular protease activity of *Pseudomonas fluorescens* (*** *p* < 0.001), where the CK group is the positive control group.

Figure 5 illustrates that, on the skim milk plate, the addition of the supernatant from untreated *Pseudomonas fluorescens* resulted in a halo. This indicates that the protease secreted by the bacteria degraded the protein present on the plate, leading to the formation of this halo. When the concentration of the CFS is 1 mg/mL, the extracellular protease activity falls below 10%. However, with an increase in CFS concentration to 2 mg/mL, there is a slight rise in extracellular protease activity. This could potentially be attributed to the leakage of intracellular proteases due to extensive bacterial death, although this effect is not statistically significant. These findings suggest that the CFS can inhibit the production of extracellular proteases by *Pseudomonas fluorescens*.

**Figure 6:**
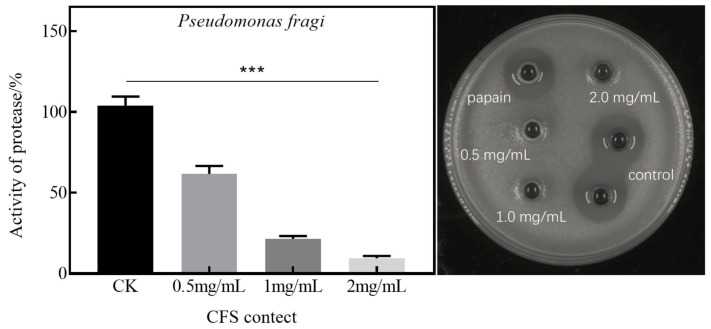
The effect of the CFS on the extracellular protease activity of *Pseudomonas fragi* (*** *p* < 0.001), where the CK group is the positive control group.

Figure 6 illustrates that, upon the addition of the untreated bacterial supernatant (wells 5–6) to the skim milk plate, a halo was formed. This suggests that the protease secreted by the bacteria degraded the protein in the plate, leading to the formation of the halo. Figure 6 reveals that at a CFS concentration of 2 mg/mL, the extracellular protease exhibits virtually no activity. This indicates that *Pseudomonas fragi* is entirely inactivated by treatment with 2 mg/mL CFS [21]. The addition of the bacterial supernatant treated with the CFS results in a reduction in the diameter of the halo. Moreover, as the CFS concentration increases, so does the reduction in the diameter of the halo. This suggests that the CFS can inhibit the production of extracellular proteases by *Pseudomonas fragi*.

### 3.4. Effect of CFS on Protein and DNA Released from Pseudomonas fluorescens and Pseudomonas fragi

**Figure 7:**
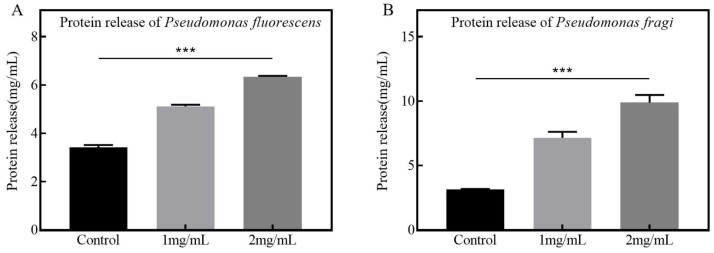
Effect of CFS on protein released from *Pseudomonas* (*** *p* < 0.001), where subfigures (**A**,**B**) refer to *Pseudomonas fluorescens* and *Pseudomonas fragi*, respectively.

The disruption of bacterial cell membranes results in the release of intracellular proteins. An increase in released proteins within the bacterial supernatant is indicative of membrane destruction. As illustrated in Figure 7A,B, at a CFS concentration of 1 mg/mL, the protein released from *Pseudomonas fluorescens* was found to be 1.3333 times that in the control group, while for *Pseudomonas fragi*, it was 2.250 times. At a CFS concentration of 2 mg/mL, the protein released from *Pseudomonas fluorescens* increased by 1.955 times, and for *Pseudomonas fragi*, it reached an impressive 3.125 times. These findings reaffirm that the CFS can induce rupture and death in both *Pseudomonas fluorescens* and *Pseudomonas fragi* by disrupting their cell membranes, thereby effectively inhibiting their growth.

**Figure 8:**
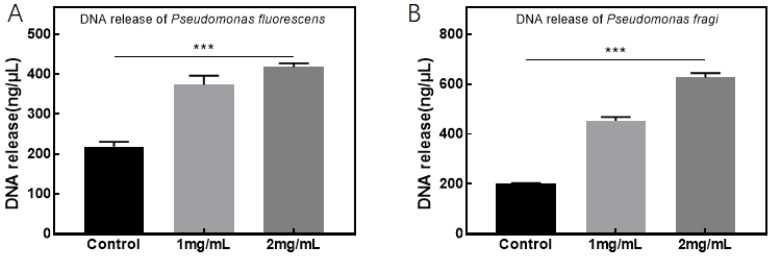
Effect of CFS on DNA released from *Pseudomonas* (*** *p* < 0.001), where subfigures (**A**,**B**) refer to *Pseudomonas fluorescens* and *Pseudomonas fragi*, respectively.

Figure 8A illustrates that the quantity of DNA released from *Pseudomonas fluorescens* markedly escalates following treatment with the CFS. Notably, when the concentration of the CFS is 2 mg/mL, the amount of DNA released surpasses 400 ng/μL. This outcome underscores the potent membrane-disrupting effect of the CFS on *Pseudomonas fluorescens*, demonstrating a dose-dependent response. Figure 8B reveals an analogous increase in the quantity of DNA released from *Pseudomonas fragi* post-CFS treatment, with the release reaching 600 ng/μL at a CFS concentration of 2 mg/mL. These findings imply that for *Pseudomonas fragi*, the membrane-disrupting impact of the CFS is more pronounced than for *Pseudomonas fluorescens*. This disparity could be attributed to the fact that the CFS also disrupts the biofilm of *Pseudomonas fragi*, thereby enhancing its membrane-disrupting effect. Conversely, the inhibition of *Pseudomonas fluorescens* may involve additional mechanisms, such as a pH reduction induced by the CFS, which results in cell shrinkage and damage to the cytoplasmic membrane, ultimately leading to bacterial death, as suggested by previous studies conducted by our research group.

### 3.5. Influence of CFS on Metabolism of Pseudomonas fluorescens and Pseudomonas fragi

**Figure 9:**
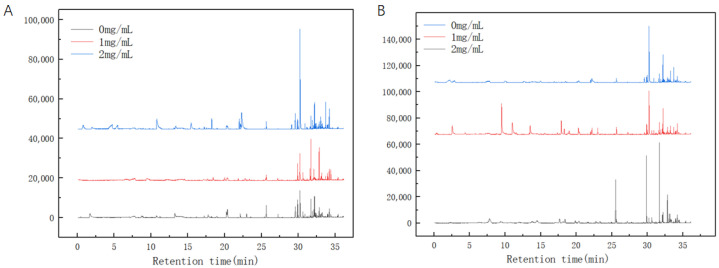
The effect of the CFS on the metabolism of *Pseudomonas*, where subfigures (**A**,**B**) refer to *Pseudomonas fluorescens* and *Pseudomonas fragi*, respectively; detailed values can be found in Supplementary Materials.

**Figure 10:**
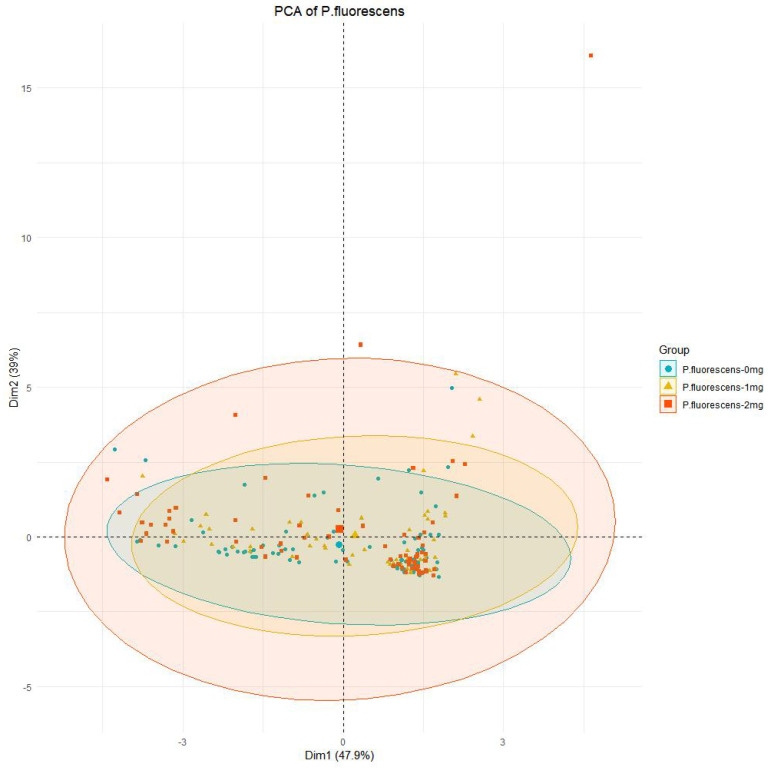
Principal component analysis of metabolites of *Pseudomonas fluorescens*.

As illustrated in Figure 9A, varying concentrations of the CFS significantly influence the metabolites of *Pseudomonas fluorescens*. In the absence of CFS, 85 substances are identified, signifying a high richness of metabolites, albeit with low peak values. This suggests that the metabolites of untreated *Pseudomonas fluorescens* are intricately complex, encompassing diverse metabolic pathways. Conversely, when the concentration of the CFS increases to 1 mg/mL, there is a notable decrease in metabolite abundance, with only 54 substances detected. Notably, substances such as 3,4-Dihydroxymandelic acid, Cyclononasiloxane, and Cyclopentanecarboxamide exhibit increased levels, while Cyclohexasiloxane demonstrates a decline. Upon further increasing the CFS concentration to 2 mg/mL, a total of 73 substances are identified, indicating an increase in abundance compared to the 1 mg/mL concentration. Substances like 3,4-Dihydroxymandelic acid and 3,6-Dioxa-2,4,5,7-tetrasilaoctane also display elevated peak values. This could potentially be attributed to the higher concentration of the CFS instigating the rupture of bacterial cell membranes, thereby leading to a continuous leakage of intracellular substances. Figure 10 shows the principal component analysis (PCA) of *Pseudomonas fluorescens* metabolites, which underwent overall structural changes after treatment with the CFS, according to the weighted distances shown in the figure. The reversal in the opposite direction was greater at 2.0 mg/mL (opposite to the 0 mg/mL group), which indicates that the metabolism of *Pseudomonas fluorescens* was greatly disturbed after CFS treatment.

**Figure 11:**
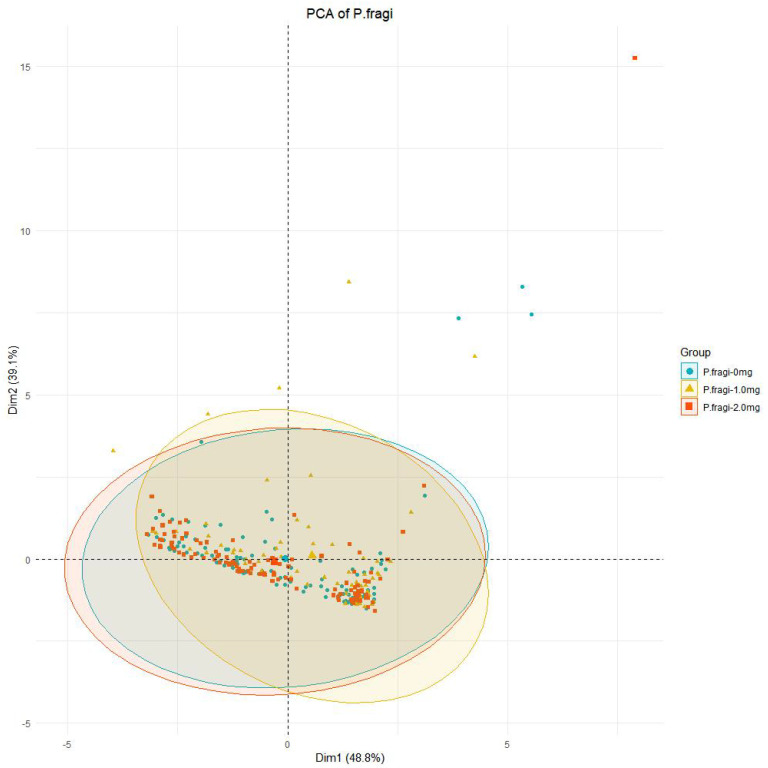
Principal component analysis of metabolites of *Pseudomonas fragi*.

As illustrated in Figure 9B, varying concentrations of the CFS significantly influence the metabolites of *Pseudomonas fragi*. In the absence of the CFS, a total of 102 substances are identified, signifying high richness and peak values of metabolites. This implies that the metabolites of untreated *Pseudomonas fragi* exhibit complexity with diverse metabolic pathways. Conversely, when the concentration of the CFS is at 1 mg/mL, there is a decrease in metabolite abundance, with only 72 substances detected. Substances such as methoxy-phenyl, Cyclononasiloxane, and Cyclopentanecarboxamide show elevated levels, while 3,6-Dioxa-2,4,5,7-tetrasilaoctane and Cyclohexasiloxane display reduced levels. When the CFS concentration reaches 2 mg/mL, a total of 115 substances are detected, indicating an increase in abundance compared to the 1 mg/mL concentration. Furthermore, the abundance of metabolites at a CFS concentration of 2 mg/mL exceeds that detected in the absence of the CFS. Figure 11 shows the principal component analysis (PCA) of metabolites of *Pseudomonas fragi*, where the distance within the circle represents the size of the difference between the groups. It can be clearly seen that the distance between the 1.0 mg/mL group and the 0 mg/mL group is the largest, where the metabolism of *Pseudomonas fragi* is disordered after treatment with 1.0 mg/mL CFS, and the distance is even smaller after treatment with 2.0 mg/mL CFS, indicating that *Pseudomonas fragi* dies before its metabolism is disordered.

## 4. Discussion

The objective of this research was to examine the constituents of CFS and its impact on the cell membrane, protease activity, protein and DNA release, and metabolic variations in the opportunistic pathogens *Pseudomonas fluorescens* and *Pseudomonas fragi*. The aim was to delve deeper into the physiological functions and antibacterial mechanisms of CFS. Different strains of lactic acid bacteria yield distinct fermentation products. Each strain produces metabolites with unique characteristics, which contribute to their varying physiological functions. To further investigate the antibacterial properties of CFS, its components were extracted using solid-phase microextraction. This was followed by GC-MS detection and database comparison, with the results presented in Table 1.

The inhibitory effect of the CFS on *Pseudomonas fluorescens* and *Pseudomonas fragi* is exerted by the organic acids and proteins it contains, which was verified in our previous study [21]. In this study, organic acids accounted for a large proportion of the 94 substances, which yielded a change in the pH gradient in the environment after CFS treatment, followed by an osmotic pressure increase and, thus, cell membrane damage. Predominantly, aside from organic acids, ketones and alkanes were the most abundant substances in the CFS. The existence of ketone compounds such as lactones suggests that the CFS has the ability to enhance food flavor and properties [24,25,26]. Furthermore, compared to the fermentation broth of conventional *Lactobacillus*, the concentrations of ketones and other organic compounds have been found to be higher in cold-adapted species [27,28,29,30]. This could potentially be attributed to their complex metabolism due to their psychrophilic characteristics, which leads to the production of more organic compounds during fermentation. In other words, in order to better adapt to harsh external conditions, some psychrophilic bacteria will speed up their metabolism and secrete special sugars and proteins to protect themselves from these conditions. These findings suggest significant potential for further development and utilization, indicating that the CFS may possess additional physiological functions.

Research examining the antibacterial properties of *Lactobacillus* fermentation broth against Burkholderia cepacia through GC-MS analysis identified 3-isobutyl-2,3,6,7,8,8a-hexahydropyrrolo[1,2-a]pyrazine-1,4-dione as a common compound, which is recognized as a potential diketopiperazine moiety. The presence of ketone substances in the CFS may also contribute to its antibacterial activity [31]. Furthermore, actinomycete-produced fermentation broth containing pyrrolo[1,2-a]pyrazine-1,4-dione has demonstrated inhibitory effects on pathogens such as *Staphylococcus aureus* (MTCC-3160) and *Pseudomonas aeruginosa* (MTCC 1688) [32].

Proteins and carbohydrates are the main substances serving physiological functions, which means that the detection of protein and carbohydrate contents in the CFS is also indispensable for predicting its physiological function [33,34]. The *Dellaglioa algida* CFS is rich in proteins, which may be caused by cold-adapting properties that promote growth metabolism. For example, certain psychrophilic bacteria can be used as one of the main sources of protease production in the food industry [35]. A carbohydrate-rich composition provides better protection for *Dellaglioa algida*, as a *Lactobacillus* species that can grow under low-temperature conditions, and allows it to adapt to the cold environment. In other words, the unique living environment promotes its production of more abundant carbohydrate substances, possibly with stronger physiological activity [36]. These primarily consist of small-molecular-weight exoproteins and extracellular polysaccharides secreted by *Lactobacillus*, which often exhibit strong physiological functions and immunoreactivity. Examples include antimicrobial peptides [37] and antibacterial polysaccharides [38], both of which are well known. It is worth noting that certain *Lactobacillus* polysaccharides can inhibit harmful bacterial biofilms. For example, six kinds of *Lactobacillus* polysaccharides extracted from children’s feces and dairy products have the effect of inhibiting the biofilms of *Bacillus cereus*, *Listeria monocytogenes*, *Enterococcus faecalis*, and *Pseudomonas aeruginosa* [39]. *Armillaria mycelium* polysaccharides can inhibit the growth of *Escherichia coli*, *Proteus*, *Bacillus subtilis*, and *Staphylococcus aureus* cells, mainly by increasing the conductivity of the culture medium and causing the overflow of intracellular substances [40]. *Lactobacillus reuteri* SHA101 polysaccharides and *Lactobacillus vaginalis* SHA110 polysaccharides isolated from the ceca of healthy chickens showed strong in vitro antibacterial activity against *Escherichia coli* and *Salmonella typhimurium* [41]. Therefore, the abundant polysaccharides in the *Dellaglioa algida* CFS may have a promoting effect on its antibacterial activity.

Ethidium bromide (EB) is capable of penetrating only cells with compromised cell membranes and emits red fluorescence when incorporated into nuclear DNA [42]. Consequently, this method can effectively ascertain whether the bacterial cell membrane has sustained significant damage. This finding holds guiding significance for investigating whether the inhibitory mechanism of the CFS against *Pseudomonas* involves membrane damage. Prior research conducted by our group has demonstrated that the minimum inhibitory concentration (MIC) of the CFS for *Pseudomonas fluorescens* and *Pseudomonas fragi* is 2 mg/mL, and it exerts a destructive effect on the biofilm of *Pseudomonas fragi* [21]. The findings of this study further substantiate that the CFS can inflict varying degrees of damage to the cell membranes of both *Pseudomonas fluorescens* and *Pseudomonas fragi*, resulting in the increased release of intracellular proteins and DNA. Furthermore, it diminishes the protease activity of *Pseudomonas fluorescens* and *Pseudomonas fragi* due to metabolic inhibition, leading to a reduction in metabolite abundance and ultimately causing extensive bacterial death, thereby exerting its antibacterial effect.

The incorporation of the CFS results in the demise of *Pseudomonas fluorescens* and *Pseudomonas fragi*, with a corresponding impact on their metabolites. To delve deeper into the antibacterial mechanism of the CFS, this research employed full-scan GC-MS analysis to scrutinize and juxtapose the substances produced after exposure to varying concentrations of CFS. By examining the disparities in metabolites, we extrapolated the potential antibacterial mechanism of the CFS. Studies analyzing volatile metabolites from bacteria during aseptic pork storage using GC-MS revealed that metabolites produced by *Pseudomonas fragi* possess significant spoilage potential. Conversely, volatile compounds linked to lactic acid bacteria are primarily alcohols and carbonyl compounds containing seven or eight carbon atoms [43]. In contrast to co-cultures in spoiled pork, Figure 9 from this study demonstrates that common substances found in spoiled meat, such as 2-ethyl-1-hexanol and esters, either decreased or were entirely undetectable in *Pseudomonas fluorescens* and *Pseudomonas fragi* treated with the CFS. This suggests that the CFS may have the potential to mitigate spoilage caused by *Pseudomonas fluorescens* and *Pseudomonas fragi*.

The above results indicate an increase in the content of 3,4-Dihydroxymandelic acid in *Pseudomonas fluorescens*, which is an intracellular synthesis product [44], indicating that the CFS impacts the cell membrane and metabolites of *Pseudomonas fluorescens* [45]. Thus, the CFS not only causes the rupture of *Pseudomonas fluorescens* cells but may also alter its metabolic capacity, thereby enhancing its antibacterial effects. In the treatment of *Pseudomonas fragi*, a higher concentration of CFS is correlated with a greater abundance of metabolites. This is likely due to the increased CFS concentration causing bacterial cell membrane rupture, leading to a continuous leakage of intracellular substances. Notably, the contents of 3,4-Dihydroxymandelic acid and 3,6-Dioxa-2,4,5,7-tetrasilaoctane were elevated, which further demonstrates that the CFS affects the cell membrane and metabolites of *Pseudomonas fragi*, leading to cell rupture and impaired metabolic capacity. Additionally, the CFS may inhibit biofilm formation, causing abnormal metabolism in *Pseudomonas fragi* and significantly increasing metabolite abundance.

## 5. Conclusions

This study provides a more nuanced understanding of the inhibition mechanism of the CFS of *Dellaglioa algida*, a newly found psychrophilic *Lactobacillus*, against *Pseudomonas fluorescens* and *Pseudomonas fragi*. By analyzing the primary components of the CFS and its effects on bacterial cell membrane damage, protease activity, protein and DNA release, and metabolite differences post-treatment, it was found that the CFS is rich in phenols and aldehydes. Both *Pseudomonas fluorescens* and *Pseudomonas fragi* exhibited varying degrees of cell membrane damage after CFS treatment, evidenced by heightened red fluorescence following EB staining and the increased release of intracellular protein and DNA. In addition, full-scan GC-MS analysis revealed that the CFS also reduced extracellular protease activity in both bacteria, resulting in significant changes in metabolite abundance. These findings clarify that the inhibitory mechanism of CFS against *Pseudomonas fluorescens* and *Pseudomonas fragi* involves its impact on their cell membranes and metabolites. This not only leads to cell rupture but also potentially impairs their metabolic capacity, further inhibiting biofilm formation in *Pseudomonas fragi* and thereby enhancing its antibacterial effects. By virtue of its antibacterial effects in cold environments, *Dellaglioa algida* shows promise in the production design of *Lactobacillus*-derived food additives, drugs, perfumes, or other chemistry-related applications. It is expected that one can establish the antibacterial spectrum of *Dellaglioa algida* and its inhibition differences among different pathogens as well as further determine the factors for the inhibitory effect of the metabolites in its CFS using HPLC or GC, which will further promote the development of *Lactobacillus*-derived products in various areas.

## Figures and Tables

**Table 1:** Analysis of composition of CFS using NIST 7 database.

Peak#	Ret.Time	Proc.From	Proc.To	Area	Height	A/H	Conc.
A	8.756	8.569	9.4	2,937,043	253,410	11.59	31.56
B	29.902	29.844	29.969	27,1905	175,282	1.55	2.92
C	30.238	30.157	30.95	4,748,302	1,802,451	2.63	51.03
D	31.724	31.682	31.788	19,9037	191,866	1.04	2.14

## Data Availability

The original contributions presented in the study are included in the article/Supplementary Materials, further inquiries can be directed to the corresponding author.

## Supplementary Materials

The following supporting information can be downloaded at https://www.mdpi.com/article/10.3390/foods13182986/s1: Table S1: Components analysis of CFS; Table S2: Components analysis of Pseudomonas fluorescens by 0 mg CFS treatment; Table S3: Components analysis of Pseudomonas fluorescens by 1 mg CFS treatment; Table S4: Components analysis of Pseudomonas fluorescens by 2 mg CFS treatment; Table S5: Components analysis of Pseudomonas fragi by 0 mg CFS treatment; Table S6: Components analysis of Pseudomonas fragi by 1 mg CFS treatment; Table S7: Components analysis of Pseudomonas fragi by 2 mg CFS treatment.
